# Modulating mitochondria with natural extract compounds: from bench to clinical therapeutic opportunities for COPD

**DOI:** 10.3389/fphar.2025.1531302

**Published:** 2025-05-21

**Authors:** Qiao Wang, Ziling Zeng, Linlin Guo, Kent E. Williams, Yun Zhang, Hongmei Tang, Hang Hu, Gang Qin, Kaijin Wang, Xing Wang

**Affiliations:** ^1^ Inflammation & Allergic Diseases Research Unit, The Affiliated Hospital of Southwest Medical University, Luzhou, China; ^2^ Department of Microbiology and Immunology, Indiana University School of Medicine, Indianapolis, IN, United States; ^3^ Indiana University School of Medicine, Melvin and Bren Simon Comprehensive Cancer Center, Indianapolis, IN, United States; ^4^ Department of Respiratory and Critical Care Medicine, The Affiliated Hospital of Southwest Medical University, Luzhou, China; ^5^ Department of Respiratory and Critical Care Medicine, Bishan Hospital of Chongqing, Bishan Hospital of Chongqing Medical University, Chongqing, China; ^6^ Department of Otolaryngology Head and Neck Surgery, The Affiliated Hospital of Southwest Medical University, Luzhou, China

**Keywords:** Mitochondria, mtROS, cigarette smoke, lung disease, natural extract compound, COPD

## Abstract

Chronic obstructive pulmonary disease (COPD) is a chronic respiratory disease that leads to death and disability worldwide, and it is caused by hereditary and environmental factors. It is characterized by chronic inflammation, emphysema, and irreversible limitation of airflow. Dual or triple therapy with a traditional approach can provide relief from COPD symptoms by reducing the frequency and severity of the outbreaks, but there are no current therapies to reverse the long-term decline in lung function. Although ICS rescue inhalers demonstrate efficacy in acute attacks, these cannot be utilized for chronic management of COPD due to adverse effects. Therefore, novel agents and therapeutic strategies are urgently needed to address this disease. It is believed that malfunctioning mitochondria are associated with COPD pathogenesis, contributing to inflammation, apoptosis, and cellular senescence. A better understanding of these mechanisms could provide novel therapeutic approaches for maintaining lung and skeletal muscle function. Many natural extract compounds show therapeutic potential for COPD and are associated with few adverse reactions. Notably, these natural compounds can improve mitochondrial function and exhibit a variety of anti-inflammatory, antioxidant, and immunomodulatory properties. In this review, we systemically summarize the pathogenic role of impaired mitochondria in COPD and the potential mechanisms by which natural extract compounds may ameliorate these impairments.

## 1 Introduction

Chronic obstructive pulmonary disease (COPD) is a chronic respiratory disease associated with heredity, the environment, individual development, and self-behavior. It is usually characterized as chronic inflammation, emphysema, and irreversible airflow limitation and causes high morbidity and mortality especially in the adult population (Doucet et al., 2016; Global Burden of Disease, 2017; Global Burden of Disease, 2018). In 2019, 212.3 million cases of COPD were reported globally, with an age-standardized point prevalence of 2,638.2 per 100,000 people, which was a decrease of 8.7% since 1990, but the absolute counts are on the rise (Safiri et al., 2022). Due to the large percentage of aging people, the number of COPD patients might increase in the coming years. Long-term exposure to cigarette smoke, air pollution, occupational dust, chemicals, biomass fumes, and other risk factors are considered risk factors that cause COPD (Agusti et al., 2023). According to the global initiative for chronic obstructive lung disease guidelines, drugs that are β2-agonists, anti-muscarinics, corticosteroids, antibiotics, antioxidants, and mucolytic are widely used to relieve COPD symptoms; additionally, the drugs can only control or delay COPD progression but cannot prevent long-term functional decrease (Agusti et al., 2023). Therefore, novel and safe medications are eagerly needed to effectively inhibit COPD progression, promote pulmonary rehabilitation, and reduce severity and mortality.

Mitochondria are responsible for a variety of cellular functions, including metabolism, intracellular signal transduction, energy production, and cell death. Previous studies have demonstrated that mitochondria play an important role in the development and progression of chronic respiratory diseases, including COPD (Yue and Yao, 2016; Zhou et al., 2021), and it might become a promising therapeutic target (Hu et al., 2022).

In this review, we will discuss COPD pathogenesis that is associated with impaired mitochondria and the potential therapeutic mechanisms by modulating mitochondrial activities with natural extracted compounds.

## 2 Mitochondrial dysregulation in COPD

COPD is a heterogeneous disease with an underlying disease process, and smoking is a high-risk factor of pathogenesis (Salvi, 2014). Cigarette smoke (CS) can impact mitochondrial structures and function of COPD epithelia after long-time exposure (Hoffmann et al., 2013). In the following sections, we will focus on the relationship between the pathogenesis of COPD and impaired mitochondrial function, such as excessive mitochondrial reactive oxygen species (mtROS), impaired mitochondrial DNA (mtDNA) and mitophagy, and impaired mitochondrial membrane potential.

### 2.1 mtROS is associated with COPD

**FIGURE 1:**
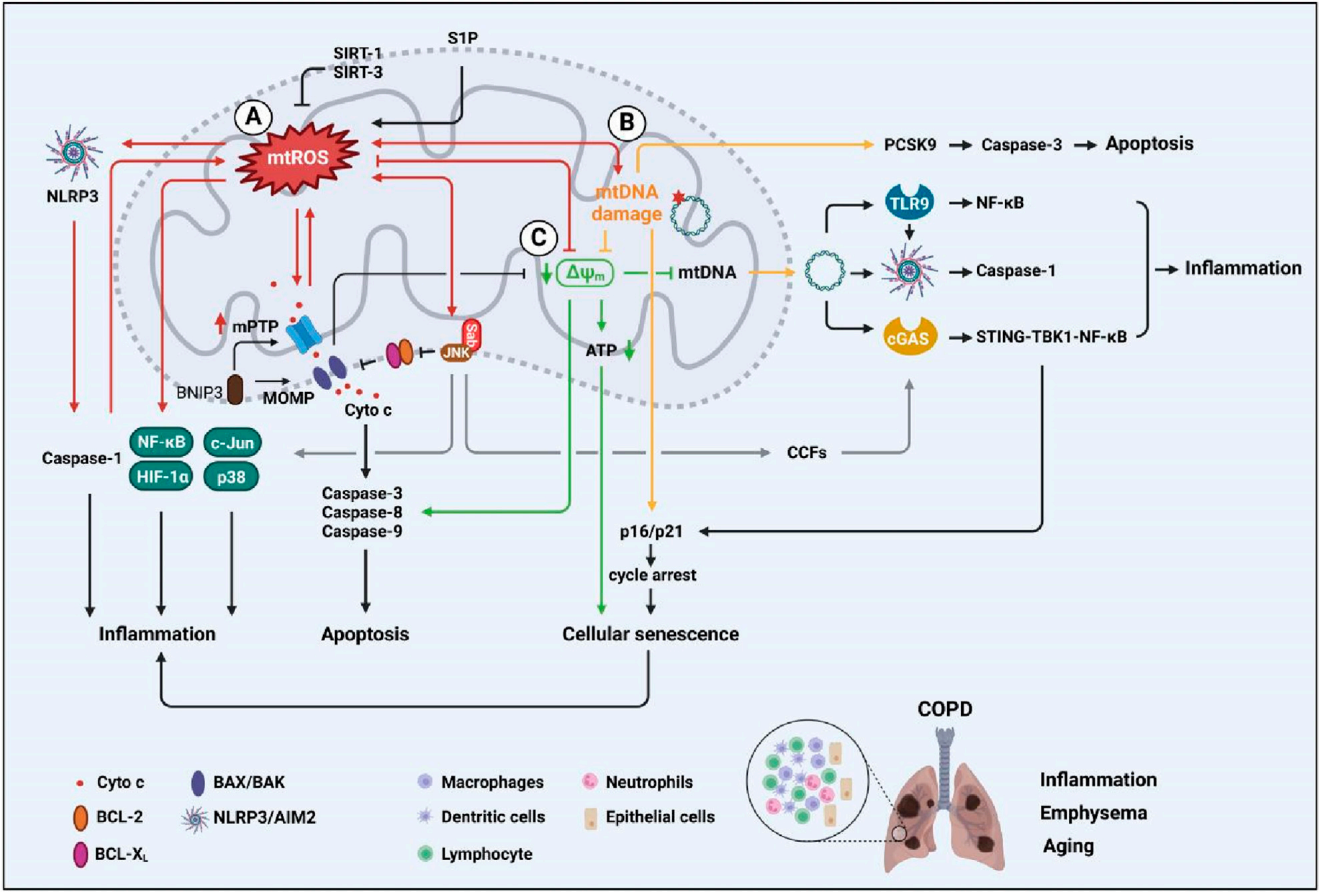
Malfunctioned mitochondrial activities are associated with cellular senescence, apoptosis, and inflammation of COPD patients. **(A)** mtROS production and its networks that are associated with inflammation, apoptosis, and cellular senescence; **(B)** mtDNA contributes to inflammatory responses and also leads to cellular apoptosis and cellular senescence; **(C)** MMP signaling is related to apoptosis, cellular senescence, and inflammation.

Dysfunctional mitochondria are highly related with pathogenesis of COPD that might via mitochondrial damage associate molecular patterns (DAMPS) from dying or stressed cells including enhanced ROS production, inflammation response as well as cellular senescence (Lerner et al., 2016; Brusselle et al., 2011). COPD shows increased oxidative stress, especially during acute outbreaks caused by the overproduction of ROS resulting from CS, air pollution, biomass smoke, inflammatory responses of lung infections, and mitochondrial stress (Noguera et al., 2001). Compared with a nonsmoker or a population that quit smoking for a long time, the bronchial epithelia from COPD patients showed enhanced mtROS and lower levels of manganese superoxide dismutase 2 (SOD2) (Haji et al., 2020). Decreased mitochondrial complexes and increased mtROS were noticed in the airway smooth muscular cells of COPD patients (Wiegman et al., 2015) (Figure 1A).

Mitochondrial deacetylase sirtuin 3 (SIRT3) is an upstream signaling factor that regulates mtROS homeostasis (Bause and Haigis, 2013). It can directly bind with and deacetylate mitochondrial antioxidant enzymes such as SOD2 (Tao et al., 2010), isocitrate dehydrogenase 2 (Yu et al., 2012), and glutathione peroxidase (Liu et al., 2015), resulting in the increased activity to regulate the mtROS level (Bause and Haigis, 2013). In contrast, the overexpression of SIRT3 leads to significant abolishing of trimethylamine N-oxide (TMAO)-induced SOD2 suppression and mtROS production (Zhang et al., 2017). The study suggested that curcumin might be involved in the upregulation of PGC-1α/SIRT3 signaling to attenuate impairing skeletal muscle mitochondria in COPD rats (Zhang et al., 2017). In addition, high-level SIRT3 can prevent SOD2 from decreasing and can also improve the mitochondrial oxidative stress of cigarette smoke extract (CSE)-treated airway epithelia, which suggests that SIRT3 might contribute to the suppression of COPD pathogenesis by inhibiting mitochondrial oxidative stress *via* the modification of SOD2 (Zhang et al., 2020). Another family member, namely, SIRT1, is involved in the regulation of mitochondrial oxidative stress gene expression, including NOX4 (Dasgupta et al., 2020) and SOD2 (Liu et al., 2019). SIRT1 overexpression can protect airway epithelia from CS-induced senescence by interacting with FOXO3; it suggests that modulating mitochondrial function might be a potential therapeutic strategy for COPD treatment (Yao et al., 2012; Aghapour et al., 2020).

#### 2.1.1 mtROS and inflammation

CS poses the risk of COPD that is highly associated with ROS production (Feng et al., 2023). Macrophages play a critical role in the iron homeostasis, and impaired macrophage leads to ROS overproduction that is associated with COPD development (Belchamber et al., 2019). mtROS might affect macrophage M1 polarization *via* MAPK, JNK/c-Jun, JNK-SOD2 and JNK-m6A-p38 signaling pathways in COPD (Feng et al., 2023; Hu et al., 2023; Liu Z. et al., 2020;). It is indicated that the airway epithelium produces more mtROS with aging, which leads to accelerated lung aging in COPD patients (Chen Q. et al., 2023). S1P can induce oxidative stress and NLRP3 inflammasome activation, leading to lung injury (Gong et al., 2023). These results indicate that S1P might affect macrophage polarization by inducing mtROS generation in COPD. It shows that the mtROS inhibitor, mitoTEMPO, can reduce NLRP3 expression in lung tissue (Zhao et al., 2018). In turn, activated NLRP3 inflammasomes can amplify mitochondrial damage *via* caspase-1, which is manifested by increasing mtROS production, dissipation of mitochondrial membrane potential (MMP/ΔΨm), loss of outer and inner membrane integrity, and fragmentation of the mitochondrial network (Yu et al., 2014). Other than promoting inflammatory response, the NLRP3 inflammasome can activate caspase-1 to induce emphysema as well; this process is independent of mtROS (Zhao et al., 2018). Correspondingly, COPD, especially in airway epithelia and macrophages, was found to increase activated NF-κB and NLRP3 inflammasome (Le et al., 2020; Rumora et al., 2021). The activated NLRP3 inflammasome-associated pyroptosis that promotes COPD pathogenesis was identified in CS-induced COPD mouse as well as COPD patients (Wang L. et al., 2021). The mtROS production and hypoxia-inducible factor-1 alpha (HIF-1α) stabilization is a marker of pro-inflammatory macrophage activation (Willenborg et al., 2021). The mtROS production has been shown to cause DNA damage, unfolded protein response, and inflammatory responses through the HIF-1α and MAPK/NF-κB pathways in LPS-stimulated macrophages (Wang Y. et al., 2021). Rapamycin negatively regulates macrophage activation by inhibiting mtROS production and limiting the activation of NLRP3 inflammasomes (Ko et al., 2017). mtROS is critical in the COPD pathogenesis and development, by targeting mtROS might provide novel treatment strategies against COPD (Figure 1A).

Neutrophils from patients with COPD have enhanced chemotaxis, extracellular proteolysis, and overproduction of ROS compared with those from nonsmokers and healthy smoker controls (Vernooy et al., 2002). It indicated that mtROS is involved in the oxidative burst and in degranulation of human neutrophils induced by the chemoattractant fMLP *in vitro* (Vorobjeva et al., 2017). Mitochondrial permeability transition pore (mPTP) in human neutrophils is a critical step for increasing mtROS production (Vorobjeva et al., 2020). Therefore, the drugs that target mtROS may reduce neutrophil inflammation in COPD.

During COPD progression, dendritic cells (DCs) participate in the activation of other immune cells, immune tolerance, and facilitating lung remodeling (Bu et al., 2020). Following *Aspergillus fumigatus* infection, it was observed that lung leukocytes over generated mtROS, including monocyte-derived DC (Shlezinger and Hohl, 2021). Bone marrow-derived DCs stimulated by chitin-derived polymer deacetylation activated cGAS-STING-mediated type-I interferon (IFN-I) response and NLRP3 inflammasome activation by enhancing mtROS production (Turley et al., 2021). When plasmacytoid dendritic cells (pDCs) were simultaneously exposed to hypoxia and toll-like receptor 9 (TLR9) agonist, pDCs released CXCL4 that was dependent on the overproduction of mtROS (Ottria et al., 2022). However, the molecular mechanism of mtROS in DCs from COPD is not clear, and more studies are needed for clarity.

#### 2.1.2 mtROS affects lung cell apoptosis in COPD

Many studies indicate that the apoptosis of lung structural cells and pulmonary vascular endothelial cells is a critical event to initiate and participate during emphysema and COPD (Sauler et al., 2019; Gogebakan et al., 2014). It is also suggested that the mice alveolar cell destruction increased after intrathoracic injection with CSE, and a large amount of apoptotic epithelia were detected in the bronchoalveolar lavage fluid (Hattori et al., 2022). It has been reported that pulmonary epithelia and endothelia apoptosis, and apoptotic factor expression are increased in COPD patients, which is closely related to the destruction of lung tissue and the development of emphysema (Hodge et al., 2005; Kasahara et al., 2001; Demedts et al., 2006). Smoke treatment resulted in increased levels of proapoptotic proteins in the terminal bronchiolar region of rat lung tissue. Mitochondrial dysfunction has also been reported in skeletal muscle cells from COPD patients, including decreased mitochondrial density and biogenesis, and increased mtROS production, which is closely related to muscle dysfunction (Meyer et al., 2013). It has been shown that the accumulation of mtROS regulates the mitochondrial intrinsic apoptosis pathway (Yee et al., 2014) (Figure 1A).

Excessive mtROS induces the opening of mPTPs, nonspecific protein complex channels between the inner and outer mitochondrial membranes (Halestrap, 1999), resulting in the transportation of ions and metabolites from the mitochondria into the cytoplasm, leading to increasing colloidal osmotic pressure within the mitochondrial matrix, loss of membrane potential, uncoupling of oxidative phosphorylation, termination or depletion of ATP synthesis, eventual cell necrosis, *etc*. (Bernardi et al., 1999). Cyto c binds to Apaf-1 and caspase-9 in the cytoplasm to form an apoptosome complex, resulting in triggering a caspase-dependent apoptotic cascade (Li et al., 1997). Mitochondrial damage is exacerbated in results from the depletion of Cyto c, further resulting in reduced mtROS accumulation and ATP production (Green and Reed, 1998). Conversely, sesamin can increase MMP and reduce lung epithelia apoptosis by inhibiting TNF-α/IL-4-induced mtROS production (Bai et al., 2022). In addition, mtROS-activated NLRP3 inflammasome is involved in the process of apoptosis, which can increase both the BAX/Bcl-2 ratio and the cleaved caspase-3 expression level (Lin Z. et al., 2018). This suggests that mtROS is highly associated with apoptosis that is involved in the regulation of COPD development.

Overproduction of mtROS results in sustained activation of JNK signaling (Kamata et al., 2005). Mitochondrial JNK is one of the key elements in cytochrome c (Cyto c) release and activating caspase (caspase-8, caspase-9, effector caspase-3, and caspase-7) (Kamata et al., 2005; Salehi et al., 2002); moreover, the processing of Cyto c release is independent of the permeability transition of the inner mitochondrial membrane (Schroeter et al., 2003). Furthermore, JNK can catalyze the phosphorylation of Bcl-2 and Bcl-x(L) to promote apoptosis as well (Schroeter et al., 2003).

#### 2.1.3 mtROS affects lung cell senescence in COPD

Senescence-associated secretory phenotype (SASP) is a basic characteristic of senescent cells, including secreting a large number of cytokines, chemokines, matrix metalloproteinases, and growth factors into the tissue microenvironment (Kuilman et al., 2008; Acosta et al., 2008), which are associated with the development of COPD. COPD-derived fibroblasts secrete higher levels of parts of SASPs (Kuilman et al., 2008; Acosta et al., 2008). Senescent lung cells secrete COPD-associated SASPs, such as IL-6, IL-8, monocyte chemoattractant protein-1 (MCP-1), and plasminogen activation inhibitor-1 (PAI-1), which further promote chronic inflammation in COPD (Freund et al., 2010). COPD is considered a disease that accelerates lung aging, and it is associated with features of cellular senescence, DNA damage, oxidative stress, and extracellular matrix remodeling (Ito and Barnes, 2009). Increased expression of p21^CIP1/WAF1^, p16^INK4a^, and senescence-associated β-galactosidase were found in bronchial epithelia and macrophages from healthy smokers (Tomita et al., 2002; Tsuji et al., 2006; Sundar et al., 2018). Emphysematous lungs also show an increased expression of p16, p19, and p21 (Tuder et al., 2012). Senescent cells accumulate in the lung and lose their regenerative capacity, thus limiting cellular repair and renewal in COPD lungs, which leads to the progression of emphysema and worsening lung function (Tsuji et al., 2006).

mtROS is involved in cellular senescence and is a major determinant of aging (Chen et al., 1995; Dai et al., 2012) (Figure 1A). ROS accumulation has been reported to trigger the p53/p21^CIP1/WAF1^ and p16^INK4A/Rb^ pathways, leading to irreversible cell-cycle arrest in senescent cells (Ziegler et al., 2015). The accumulation of mtROS leads to mitochondrial dysfunction and promotes cellular senescence (Hekimi et al., 2011), which is mainly dependent on mitochondrial oxidative damage, such as mtDNA mutation and mitochondrial membrane permeability. Cytoplasmic chromatin fragments (CCFs) are chromatin fragments released into the cytoplasm by senescent cells *via* budding formation from the nucleus (Ivanov et al., 2013). CCF is a trigger of SASP, and the elevated mtROS induce CCF formation in senescent cells by promoting JNK activation (Vizioli et al., 2020). DNA fragments in CCFs of senescent cells activate the cytoplasmic DNA-sensor cGAS to generate the second messenger cGAMP; cGAMP binds and activates the adapter protein STING (Wu et al., 2013; Glück et al., 2017). The cGAS-STING signaling promotes TBK1 recruitment and phosphorylation, resulting in IRF3 nucleus translocation and enhanced IFN-I expression. On the other hand, NF-κB is activated by TBK1, leading to upregulation of type-I interferons and inflammatory cytokine expression (Yang et al., 2017). Similarly, inhibiting cGAS-STING signaling can effectively inhibit both SASP expression and astrocyte senescence (Aguado et al., 2021). Furthermore, the AMPK–mTOR pathway modulates NF-κB transcriptional activity to regulate the translational levels of pro-inflammatory factors in SASPs (van Vliet et al., 2021). TLR2, which acts as a downstream factor of STING to regulate NF-κB, was shown to be required for increasing SASP secretion *in vivo* (Hari et al., 2019).

Therefore, drugs targeting mtROS might promote lung recovery and delay emphysema changes by reducing pulmonary oxidative stress, delaying cellular senescence, and inhibiting inflammatory responses.

### 2.2 Damaged mtDNA is associated with COPD, inflammation, lung aging, oxidative stress, and cell apoptosis

mtDNA is susceptible to oxidative damage due to its proximity to ROS production sites, special packaging structures, and asymmetric replication (Reyes et al., 1998; Parisi and Clayton, 1991). Excessive mtROS results in mtDNA site mutations, insertions, deletions, and copy number reduction (Quan et al., 2020). Different types of damage to mtDNA can also occur because of endogenous and exogenous (tobacco smoke, chemicals, *etc*.) noxious substances (Alexeyev et al., 2013). Damaged mtDNA fragments can escape into the cytoplasm or extracellular space, which may be related to changes in mitochondrial permeability, mitochondrial dynamics, mitophagy, BAX/BAK pores, and VDAC1 oligomers (Pérez-Treviño et al., 2020). It has been reported that the mtDNA content does not change in end-stage COPD lung tissue, but the mtDNA strand breaks and/or untrustworthy sites are significantly increased compared with normal lung tissue (Pastukh et al., 2011). In a SPIROMICS cohort study (NCT01969344), higher levels of plasma-mtDNA were found in subjects with mild or moderate COPD than in nonsmokers and smokers without airflow obstruction (Zhang W. Z. et al., 2021). Similarly, plasma levels of both cell-free mtDNA (cf-mtDNA) and nuclear DNA (cf-nDNA) were elevated in former COPD smokers compared with that in former smokers without COPD (Giordano et al., 2022). Increased cf-mtDNA copy number was significantly associated with the development of COPD. They also found that bronchial epithelia exposed to CSE led to the release of mtDNA into the extracellular space through vehicles and cellular debris (Giordano et al., 2022).

The mtDNA released from mitochondria is considered a damage-associated molecular pattern (DAMP) by activating downstream pro-inflammatory signaling cascades (Fang et al., 2016). Activation of human polymorphonuclear neutrophils (PMNs) by mtDNA in the plasma through TLR9 induces secondary IL-8 release and activates PMN p38 and p44/42 MAPKs, leading to systemic inflammation (Zhang et al., 2010). Cytosolic mtDNA also mediates inflammatory responses in the intracellular space by contributing to IL-1β and IL-18 secretion through the activation of the NLRP3/AIM2-caspase-1 pathway (Nakahira et al., 2011). The accumulation of mtDNA activates cGAS-STING signaling and IFN-I responses in DCs, B cells, and natural killer cells (West and Shadel, 2017), resulting in enhanced inflammatory responses. In addition, damaged mtDNA can increase the expression of pro-protein convertase subtilisin/kexin type 9 (PCSK9) and elevated PCSK9 can induce apoptosis by stimulating caspase-3 (Ding et al., 2016).

Mitochondrial dysfunction plays a central role in the aging process, and mtDNA mutations are critical hallmarks of cellular aging and other related diseases (Szczepanowska and Trifunovic, 2017; van der Rijt et al., 2020) (Figure 1B). As previously described, oxidatively damaged mtDNA results in reduced gene expression of mtDNA, defects in the oxidative phosphorylation system, and increased oxidative flux (Wallace, 1992); this is a common feature in many human aging tissues, including lung tissue (Bender et al., 2006). Studies have demonstrated that mtDNA drives cGAS-dependent responses and enhances senescence in lung epithelia and fibroblasts (Schuliga et al., 2021; Schuliga et al., 2020). mtDNA mutations also accelerate oocyte aging by reducing the NADH/NAD^+^ redox state (Yang et al., 2020). The accumulation of mtDNA damage has also been shown to promote apoptosis by increasing c-caspase-3 and Cyto c release and leading to mitochondrial outer membrane permeabilization (MOMP) (Kujoth et al., 2005). These studies suggest that drugs that protect mtDNA may have the potential effect of slowing COPD progression by affecting cellular senescence and inflammatory pathogenesis (Figure 1B).

### 2.3 Disturbed mitochondrial membrane potential is associated with COPD, inflammation, and apoptosis

Mitochondrial membrane potential (MMP/ΔΨm) plays an important role in maintaining mitochondrial function, mitochondrial permeability, mitochondrial viability, and other cellular functions (Bagkos et al., 2014; Gottlieb et al., 2003). It has been observed that CSE leads to the loss of cellular ATP and rapid depolarization of MMP (Wu et al., 2020). Reduced MMP/ΔΨm was noticed in mitochondria isolated from bronchial biopsies of COPD patients (Haji et al., 2020). Human airway smooth muscle cells from COPD patients have reduced MMP, ATP content, and basal and maximal respiration compared to those from healthy controls (Wiegman et al., 2015). Likewise, both MMP and ATP levels were significantly reduced in quadriceps muscle cells of CS-exposed rats compared to controls, which are associated with the decreased physical ability of COPD rats (Mao et al., 2019). Decreased or depolarized MMP is considered an important indicator of mitochondrial dysfunction, with profound effects on inflammatory responses and apoptosis (Zhou et al., 2021) (Figure 1C).

Disrupting MMP directly affects the electron transport chain (ETC) that in turn leads to loss of oxidative phosphorylation and increases mtROS production. Depolarized MMP induces a transition in mitochondrial membrane permeability that leads to the release of mtROS, mtDNA, and intermembrane proteins into the cytosol, resulting in triggering inflammatory and proapoptotic responses (Bronner and O'Riordan, 2016). Improving mitochondrial function and decreasing mtROS generation can attenuate inflammatory response (Chen Y. et al., 2023). Cleaved oxidized-mtDNA promotes NLRP3 inflammasome and cGAS-STING signaling activation, leading to the pathogenesis of chronic inflammatory diseases (Xian et al., 2022).

MMP reduction is a key event in the induction of apoptosis (Zaib et al., 2022). The pro-apoptotic Bcl-2 family protein BAK/BAX can regulate the MMP change; the activation and oligomerization of BAK/BAX can induce homomultimeric pores formation (Korsmeyer et al., 2000) or induce the MOMP (Kuwana et al., 2002). BAK/BAX can bind to and promote the opening of VDAC1, leading to decreasing MMP (Shimizu et al., 1999). The decline in MMP causes structural changes in mitochondria, including matrix condensation and cristae disintegration (Gottlieb et al., 2003). These structural changes result in the release of Cyto c into the intermembrane space and cytoplasm (Gottlieb et al., 2003). The released Cyto c results in apoptosis by activating Apaf-1-caspase-9 apoptosome and subsequently activating executioner caspase-3 (Yuan and Akey, 2013). The impact of abnormal MMP on the pathogenesis of COPD may be more complicated, and further research is needed.

### 2.4 Impaired mitophagy is associated with inflammation and senescence in COPD

Mitophagy-associated signals are highly related to the pathogenesis of COPD, such as AMPK signaling. Studies have shown that AMPK can directly phosphorylate the serine/threonine kinase ULK1 (Kim et al., 2011; Egan et al., 2011); then, ULK1 promotes mitophagy by promoting phosphorylation of the Parkin ACT domain (Hung et al., 2021). ULK1 can also directly phosphorylate mitophagy receptors, including FUNDC1 (Wu et al., 2014), BNIP3 (Poole et al., 2021), NIX, and Bcl-2-L-13 (Kim et al., 2011; Egan et al., 2011; Murakawa et al., 2019). AMPK can induce mitophagy by regulating MFN2 to respond to energy stress (Hu et al., 2021). Increased mTOR activity leads to decreased molecular activity of PINK1/Parkin and LC3 (Wen et al., 2020). In addition, AMPK inhibits mTOR signaling by phosphorylating the mTOR factors TSC2 and Raptor (Ha et al., 2015).

SIRT1 was shown to induce mitophagy and alleviate mitochondrial damage (Biel et al., 2016). Adiponectin reduces mtROS production and inhibits the generation of inflammatory cytokines TNF-α and IL-6 *via* mitophagy through SIRT1-PINK1 signaling (Jiang et al., 2021). The transcription factor Nrf2 is crucial in maintaining mitochondrial structure and function (Dinkova-Kostova and Abramov, 2015). The activation of Nrf2 leads to mitophagy (Dinkova-Kostova and Abramov, 2015; Gumeni et al., 2021).

BNIP3, one of the pro-apoptotic factors belonging to the Bcl-2 family, can mediate mitophagy by binding to LC3 through the LIR motif in its homodimer, which is closely related to mitochondrial function disorder and cell death (Quinsay et al., 2010; Hanna et al., 2012). Airway epithelia exposed to 7.5% CSE shows BNIP3L overproduction to promote mitophagy, resulting in enhanced mitochondrial dysfunction and cellular damage (Zhang et al., 2019). Patients with systemic inflammatory COPD show higher BNIP3- and BNIP3L-mediated mitophagy marker levels than those with normal COPD, which suggests that BNIP3-mediated mitophagy might promote systemic COPD inflammation (Leermakers et al., 2018).

The activated AMPK, ULK1, and SIRT1 signaling pathways contribute to enhance mitophagy that is highly associated with emphysema, aging, and inflammatory responses. This suggests that targeting mitophagy networks may improve the physical ability and life quality of COPD patients.

#### 2.4.1 Mitophagy affects inflammation

Mitophagy has a complex relationship with COPD progression, and PINK1/Parkin signaling is considered to be a key pathway for mitophagy (Yao et al., 2021). A study found that Parkin protein expression levels were significantly lower in COPD lungs than in the lungs of nonsmokers and non-COPD smokers, and they were positively correlated with the percentage of FEV1/FVC (Ito et al., 2015).

Additional studies indicate that Parkin/PINK1-deficient mice show elevated circulating mtDNA that induces inflammatory phenotype cytokine and chemokine (IL-6, IL-12, IL-13, IFN-β, CXCL1, CCL2, and CCL4) expression by activating the cGAS–STING pathway; Parkin and PINK1 mediate mitophagy mitigating STING-induced inflammation (Sliter et al., 2018). COPD patients had higher accumulation of ubiquitinated proteins and p62 in lung homogenates than heavy smokers and light/nonsmokers, which was possibly due to insufficient CSE-induced autophagy in damaged cells (Fujii et al., 2012). Furthermore, a significant increase in the number of total cells and macrophages was observed in the bronchoalveolar lavage fluid of Parkin KO mice compared with wild-type mice exposed to CS, and Parkin plays a critical role in regulating mitophagy during COPD pathogenesis (Araya et al., 2019).

**FIGURE 2:**
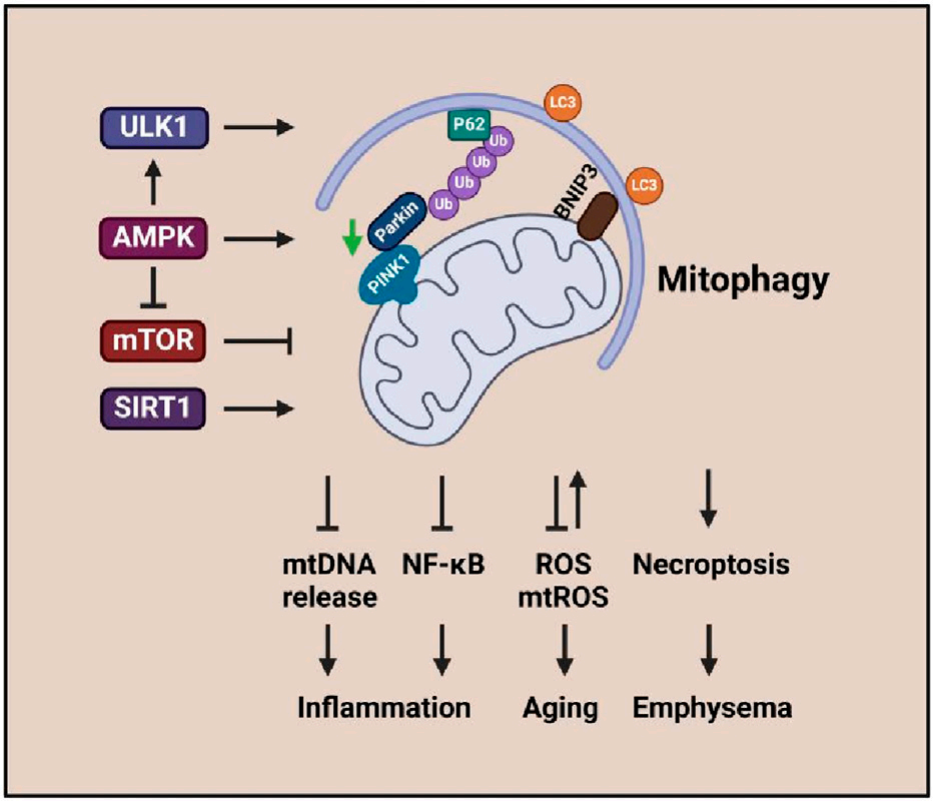
Mitophagy signaling associated with inflammation, aging, and emphysema. The enhanced ULK1, AMPK, and SIRT1 activities promote mitophagy that leads to emphysema, aging, and inflammatory response in COPD.

These results suggest that PINK1/Parkin, cGAS–STING pathways, and mitophagy regulations are essential during the pathogenesis of COPD. Sufficient and effective mitophagy is necessary and critical to maintain mitochondrial homeostasis (Figure 2).

#### 2.4.2 Mitophagy affects senescence

Emphysema and chronic bronchitis are the two major forms of COPD. Malfunctioning mitochondria and cellular senescence are associated with CS-induced pathogenesis of COPD/emphysema linked with mitophagy and PINK1/Parkin signaling (Wang et al., 2022; Ahmad et al., 2015) (Figure 2). CSE-induced PINK1-dependent mitophagy aggravates mitochondrial damage and induces necroptosis, leading to enhanced emphysema (Mizumura et al., 2014).

Imbalanced low-level mitophagy is one of the major factors that cause senescence (Figure 2). PINK1 and Parkin are involved in the pathogenesis of COPD *via* regulating mitophagy, but the effects are controversial. Studies indicate that insufficient mitophagy can result in accelerated aging of CSE-exposed human small airway epithelia (Ito et al., 2015; Ahmad et al., 2015), which is associated with cytoplasmic p53 inhibiting Parkin mitochondrial translocation (Ahmad et al., 2015). Reduced Parkin level-induced insufficient mitophagy can result in ROS overproduction and promotion of branchial epithelial aging (Ito et al., 2015). Mice deficient in Parkin showed changes in emphysema and aggravated airway wall thickening exposure to CS; impaired mitochondrial accumulation, enhanced mtROS, and increased senescence were found in bronchial epithelia (Saito et al., 2019). In contrast, pirfenidone can promote mitophagy and attenuate CSE-induced cellular senescence by inducing Parkin expression (Saito et al., 2019). Additionally, Parkin-mediated mitophagy deficiency-induced mtROS overproduction promotes the development of COPD-associated muscle atrophy (Ito et al., 2022). Elevated PINK1 represents the accumulation of impaired mitochondria, which is conferred by insufficient mitophagic degradation (Hoffmann et al., 2013).

Homeostasis of mitophagy is highly associated with senescence, which plays a critical role in the pathogenesis of COPD. Targeting mitophagy-associated senescence signaling might provide novel therapeutic strategies against COPD.

### 2.5 Mitochondrial biogenesis is associated with COPD cell aging and inflammation

Mitochondrial biogenesis plays an important role in maintaining cellular homeostasis when stimulated by environmental factors (Popov, 2020). The mTORC1/PGC-1β signaling pathway is a master regulator of mitochondrial biogenesis in mammalian cells (Correia-Melo et al., 2016). It has been found that aging lung epithelia exhibit increased mTOR/PGC-1α/β activation, which is associated with upregulation of mitochondrial biogenesis, oxidative phosphorylation, mtROS overproduction, and induction of cellular senescence (Summer et al., 2019). The activation of mTOR induces senescence of lung cells and mimics COPD lung changes with rapid development of emphysema, pulmonary hypertension, and inflammation *via* phosphorylated GSK3 and Akt^ser473^ signaling (Houssaini et al., 2018). In addition, the reduced lamin B1 in airway epithelia results in the decreasing expression of DEPTOR (a natural negative regulator of mTOR kinase activity) that is involved in aberrant activation of mTOR in response to CSE (Saito et al., 2019). Correspondingly, rapamycin reversed cellular senescence by reducing the activity of mTORC1/PGC-1α/β signaling, and rapamycin suppressed the expression of inflammatory cytokine SASPs (Summer et al., 2019; Walters et al., 2016). COPD patients show low mitochondrial biogenesis due to attenuated PGC-1α and TFAM (Taivassalo and Hussain, 2016). The increased PGC-1α suggests host cells enhancing mitochondrial biogenesis through up taking foreign mitochondria. This mitochondrial transfer can reverse mitochondrial malfunction in COPD (Frankenberg Garcia et al., 2022). Improving mitochondrial biogenesis to enhance mitochondrial function and muscle performance might lead to promising therapeutic strategies for COPD patients.

PGC-1α can interact with nuclear respiratory factors (Nrf1 and Nrf2) (Wu et al., 1999), members of the nuclear receptor family (such as PPARα, PPARγ, and ERR-1α), non-nuclear receptor transcription factors (such as myocyte enhancer factor-2 and MEF-2), and the FOXO family to regulate the expression of mitochondrial genes and mitochondrial transcription genes (TFAM, TFB1M, and TFB2M), leading to controlling the transcriptional activity of energy metabolism by key metabolic factors, namely, AMPK, SIRT1, and PGC-1α (Cantó and Auwerx, 2009; Gleyzer et al., 2005; Rizk et al., 2023). In addition, Parkin regulates PGC-1α at the transcriptional level, and they also interact with each other to regulate mitochondrial mass and function (Zheng et al., 2017). SIRT1 can directly interact with PGC-1α and deacetylate PGC-1α (Rodgers et al., 2005) to activate its downstream targets such as Nrf1 and TFAM to enhance mitochondrial biogenesis (Tian et al., 2019; Ye et al., 2022). *In vitro* SIRT1 protein levels have been reported to decrease in lung epithelia (Yao et al., 2012), endothelial cells (Arunachalam et al., 2010), and macrophages after exposure to CSE; this finding was also noticed in the lungs of smokers and patients with COPD (Rajendrasozhan et al., 2008). CS mediates pro-inflammatory responses and senescence that is associated with impaired SIRT1/FOXO3 signaling (Yao et al., 2012; Di Vincenzo et al., 2018; Yao et al., 2014). Hesperidin inhibits CSE-induced inflammatory response in a COPD rat model *via* modulating SIRT1/PGC-1α/NF-κB signaling (Manevski et al., 2020). In addition, SIRT1 activators inhibit TGF-β1-mediated phosphorylation of Smad3, resulting in attenuating the CSE-mediated airway remodeling of bronchial epithelia (Guan R. et al., 2020). Therefore, SIRT1 regulates PGC-1α activity at least partly *via* TGF-β signaling. However, the specific mechanism through which the SIRT1/PGC-1α axis regulates mitochondrial activities in COPD is yet to be known, and we need more evidence to verify the mechanism.

AMPK expression is reduced in bronchial epithelia of emphysema and CSE-exposed mice (Cui et al., 2018). Prophylactically used AMPK activators can reduce inflammation and cellular senescence in emphysema mice, which has been associated with the upregulation of mitochondrial proteins including SOD2 and SIRT3; AMPK-α1/α2 knocked down in human bronchial epithelia increases cellular senescence-related gene expression (Cheng et al., 2017). Studies have shown that mitochondrial biogenes is related with SIRT3 (Zhang et al., 2017), p-AMPK-, and TFAM (Remels et al., 2007) levels. The results indicated that they are decreasing in the skeletal muscle of COPD rats model. These findings suggest the influence of AMPK on lung inflammation and aging. However, the insight mechanism of whether AMPK signaling regulates PGC-1α in COPD needs to be verified.

**FIGURE 3:**
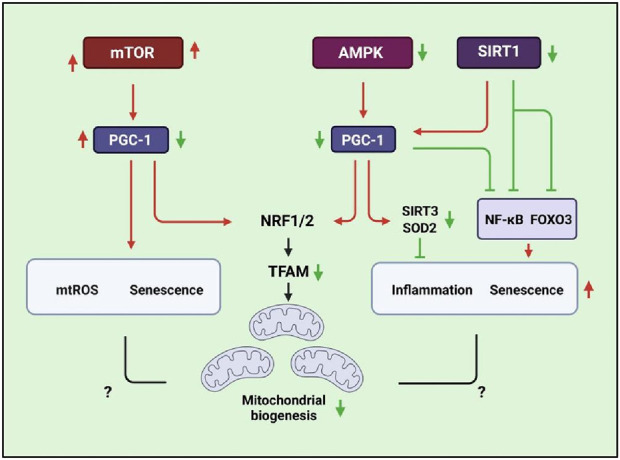
Schematic signaling indicating that mitochondrial biogenesis is associated with SIRT1, AMPK, mTOR, and PGC-1/TFAM pathways that are related to inflammatory responses, ROS production, and senescence.

Taken together, the elevated mitochondrial biogenesis is beneficial for reversing the pathogenesis of COPD. Mitochondrial homeostasis is critical for maintaining cellular survival and function; impaired mitochondrial structure and function in various cell types may induce COPD pathogenesis. However, the mechanisms by which mitochondria regulate COPD development at different stages and cell types require deep insight investments. Therefore, it might be a great challenge for the clinical application of mitochondria-targeted therapy (Figure 3).

### 2.6 The potential role for UPR^mt^ in COPD development

Mitochondrial unfolded protein response, UPR^mt^, is a pivotal component in maintaining immune homeostasis in response to internal or external stress (Bernales et al., 2012; Inigo and Chandra, 2022). Accumulated mtROS breaks the folding proteins and unfolded protein balance, resulting in UPR (Bernales et al., 2012). Meanwhile, mtROS overproduction can trigger UPR^mt^ signaling that is shown as ATF5, SIRT3, and ERα upregulation to process antioxidative activities in response to mtROS overload (Fiorese et al., 2016; Jenkins et al., 2021; Livezey et al., 2018). It is indicated that UPR^mt^ is associated with enhancing ELF2α and JNK phosphorylation in response to an inflammatory environment (Bernales et al., 2012; Nguyen et al., 2024; Gaspar et al., 2023). It has been suggested that UPR^mt^ is associated with antioxidant defense, metabolism, inflammation, *etc*. However, the evidence showing the mechanism of how UPR^mt^ contributes to COPD is limited (Kelsen, 2016). Based on our knowledge, we hypothesize that UPR^mt^ accumulation might prevent COPD pathogenesis in the early stage, but severe UPR^mt^ might play a role in COPD progression. The potential therapeutic strategies of COPD that aim at modulation of UPR^mt^ and mtROS need in-depth investigations.

### 2.7 MAMs response to stress effects on COPD pathogenesis

Mitochondria-associated endoplasmic reticulum (ER) membranes (MAMs) serve as the bridge connecting mitochondria and ER (Zhang Y. et al., 2023; Zhang et al., 2024). MAMs play a critical role in multiple functions based on different components (Elwakiel et al., 2024), such as: 1) VDAC, and IP3R are associated with stress responses and apoptosis; 2) NLRP3 is responsible for inflammation; and 3) VDAC, IP3R, GRP75, and Mitofusin 2 (MFN2) are related with Ca^2+^ transfer to maintain homeostasis, and many others. Studies show that malfunctioning MAMs can cause extensive Ca^2+^ overloading, resulting in ER Ca^2+^ efflux into mitochondria through the VDAC1-Grp75-IP3R1 complex interacting with Cyclophilin D, which leads to disease pathogenesis (Zhang et al., 2024; Elrod et al., 2010; Baines et al., 2005). Mitochondrial dynamics-related proteins are rich in MAMs, such as MFN2, OPA1, and FIS1 (Zhang Y. et al., 2023; Elwakiel et al., 2024). Silencing or mutation of MFN2 can cause inefficient Ca^2+^ uptake, leading to health problems (Gӧbel et al., 2020; de Brito and Scorrano, 2008). Overloaded mitochondrial Ca^2+^ can further lead to mtROS, mPTP, and mtDNA release, resulting in cell death signaling (Bronner and O'Riordan, 2016; Zhong et al., 2016). Moreover, MFN2, OPA1, FIS1, *etc*. are involved in the mtROS homeostasis regulation (Lin H. Y. et al., 2018). It has been shown that MFN2 and OPA1 are downregulated in COPD, whereas increasing MFN2 and OPA1 expression can somehow attenuate oxidative stress and cellular senescence (Li et al., 2023; Maremanda et al., 2021). CSE can induce bronchial epithelial mtROS and senescence by enhancing mitochondrial FIS1 expression (Hara et al., 2013). Now, we can see that the overloaded Ca^2+^ will worsen the mtROS balance that might lead to UPR^mt^ extensive activities, which might result in unpredictable COPD development. Further understanding the relation between dysfunctional mitochondria–ER tethering proteins and mtROS overproduction, Ca^2+^ overload, and mitochondrial dynamics might provide novel therapeutic strategies of COPD.

## 3 Effects of natural compounds on mitochondria

**FIGURE 4:**
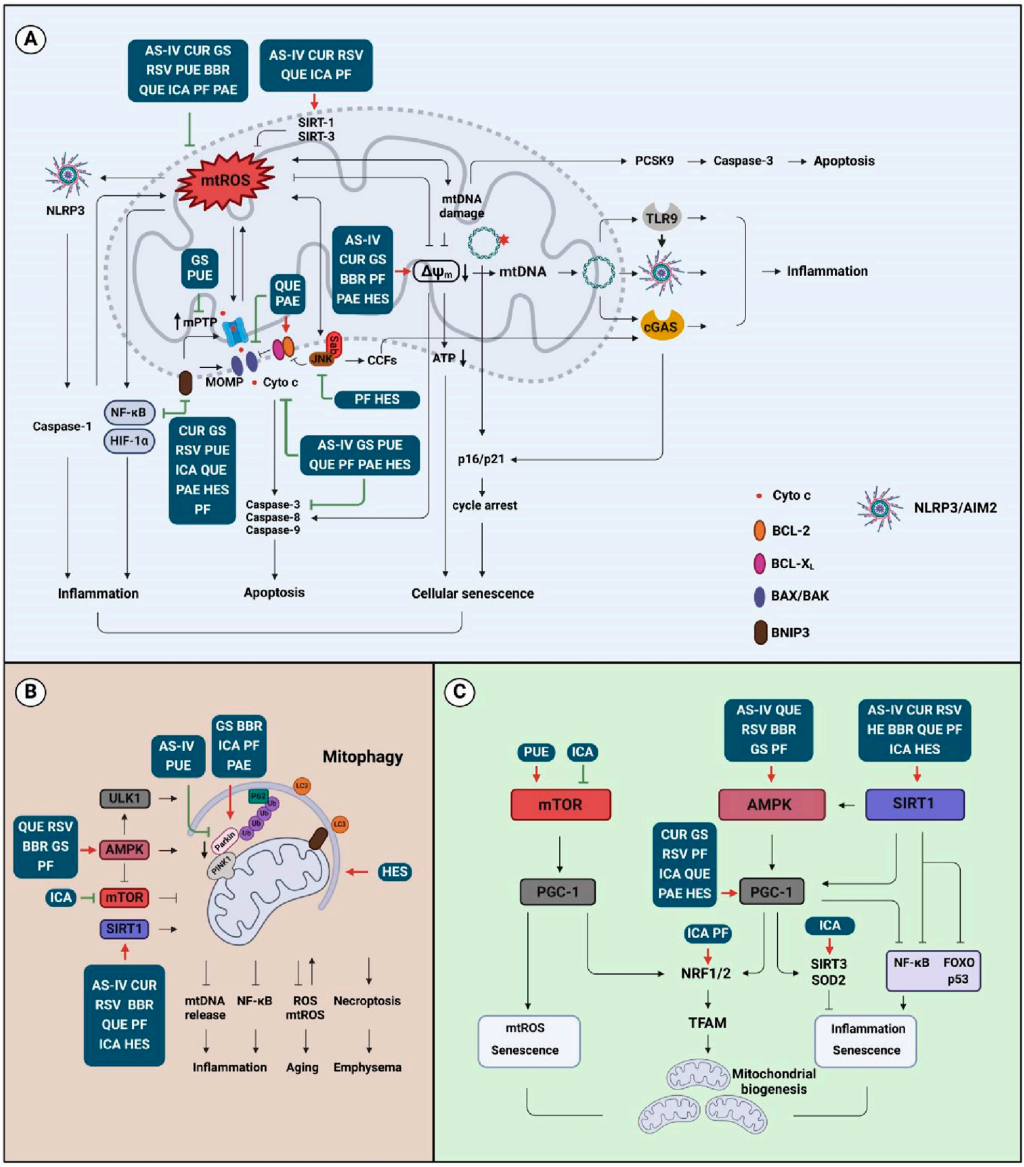
Effects of natural compounds on the mitochondrial activities. **(A)** Compound affects mtROS, mtDNA, and MMP signaling that is associated with inflammation, apoptosis, and cellular senescence; **(B)** Compound targets mitophagy-associated signaling that is related to inflammation, aging, and emphysema; **(C)** Compound regulates mitochondrial biogenesis signaling that is involved in inflammation and senescence. Abbreviation: AS-IV, astragaloside IV; BBR, berberine; CUR, curcumin; GS, ginsenosides; HES, hesperidin; ICA, icariin; PAE, paeonol; PF, paeoniflorin; PUE, puerarin; QUE, quercetin; RSV, resveratrol.

Treatments that modulate mitochondrial function are beneficial in restoring airway inflammation, promoting pulmonary recovery, and encouraging the investigations on new drugs development for COPD therapy (Manevski et al., 2020). As mitochondrial dysfunction highlights a wide range of pathological conditions in COPD, the outcome of treatment with mitochondrial-targeting drugs requires in-depth exploration. The findings revealed potential new applications from natural compounds and suggest that the regulation of mitochondria may be an important mechanism for herb extracts to treat COPD, as we discuss below (Figure 4).

### 3.1 Curcumin

Curcumin (CUR), a polyphenol and nontoxic compound obtained from turmeric (*Curcuma longa*), has been shown to exert therapeutic effects on respiratory diseases, which might be due to mitochondrial protective properties (Lelli et al., 2017). In a randomized controlled clinical study, the combination curcuminoids with piperine were shown to alleviate systemic oxidative stress and clinical symptoms in COPD patients (Panahi et al., 2016). A preclinical study indicated that CUR can improve emphysema and airway inflammation in COPD rats and reduce mitochondrial damage in alveolar epithelia (Zhang et al., 2016). CUR can also alleviate mitochondrial damage in skeletal muscle cells of COPD rats by activating PGC-1α/SIRT3 signaling (Zhang et al., 2017) and attenuate airway inflammation and airway remodeling in a COPD mouse model, which was closely related to inhibiting BEAS-2B cell proliferation and inhibiting the activation of NF-κB and cyclooxygenase-2 (COX-2) expression (Yuan et al., 2018). CUR alleviates COPD by activating SIRT1 signaling and enhancing the expression of autophagy-related proteins LC3-I, LC3-II, and Beclin-1 (Tang and Ling, 2019). These findings suggest that the mitochondrial pathway may be a potential mechanism for the treatment of COPD with CUR.

### 3.2 Ginsenosides

Ginsenosides (GS) from *Panax ginseng* (such as Rb1, Rb2, Rc, Rd, Re, Rg1, Rg3, Rg5, Rh1, and Rh2) are the main bioactive components against COPD (Chen et al., 2008; Lin et al., 2022), and ginsenosides have been found to have potential effects on mitochondrial diseases by regulating mitochondrial activities (Liu Z. et al., 2022; Zhou et al., 2019a). The anti-inflammatory effect of *ginseng* and ginsenosides in the treatment of COPD includes regulating the NF-κB pathway, and inflammatory cytokine (TNF-α, IL-6, IL-8, and IL-1β) expressions (Shergis et al., 2014).

Ginsenoside Rg3 treatment in the acute exacerbation COPD (AECOPD) mouse model resulted in decreased neutrophil migration and improvement of lung function and lung morphology (Guan X. et al., 2020). Ginsenoside Rg3 promoted mitochondrial biogenesis and increased PGC-1α, Nrf1, and TFAM levels (Lee et al., 2019). It can also promote mitophagy-related protein (LC3-II/LC3-I and Beclin-1) expressions by activating AMPK (Xing et al., 2017). In addition, ginsenoside Rg3 inhibits mPTP opening *via* scavenging free radicals to protect the mitochondrial function of nerve cells (Tian et al., 2009). Ginsenoside Rb3 might inhibit mitochondrial-associated apoptosis (Bcl-2 and BAX) and enhance energy metabolism *via* activating PPARα (Chen et al., 2019). It has been shown that ginsenoside Rd attenuates focal cerebral ischemia-mediated mitochondrial dysfunction that manifests by reducing mitochondrial swelling, maintaining MMP and respiratory chain complex activity, and reducing ROS production, Cyto c release, and apoptosis-inducing factor (AIF) expression (Ye et al., 2011). Additionally, ginsenoside Rd combined with Re can attenuate rotenone-induced mitochondrial damage that is associated with inhibiting MMP depolarization and increasing cytosolic and mitochondrial Ca^2+^ levels (González-Burgos et al., 2017). It is suggested that ginsenosides Rb1 and Rg1 can alleviate astrocyte impairment *via* reducing ROS production, increasing catalase (CAT) activity, inhibiting MMP depolarization and mtDNA content, and maintaining mitochondrial respiratory chain activity (Xu et al., 2019). Ginsenoside Rh2 promotes mitophagy *via* increasing PINK1/Parkin production (Hou et al., 2020). Notoginsenoside R1 (NGR1), a triterpenoid saponin compound extracted from *ginseng*, effectively alleviates diabetic retinopathy of db/db mice by inhibiting inflammation, reducing mtROS production, and activating PINK1-mediated mitophagy (Zhou et al., 2019b).

Increased transforming growth factor-β1 (TGF-β1) in the airway epithelium and lung cells of COPD patients promotes the recruitment of macrophages (de Boer et al., 1998; Chung et al., 2018). Ginsenoside Rg1 can not only downregulate the inflammatory response but also delay the progression of CS-induced airway remodeling by inactivating TGF-β1/Smad3 signaling (Guan et al., 2017). All these findings indicate that the properties of ginsenosides on the mitochondria are potential therapeutic strategies for COPD, and more in-depth investigations are needed to promote this natural compound to potential clinical practice.

### 3.3 Resveratrol

Resveratrol (RSV) is a natural polyphenolic phytoalexin found in many fruits and vegetables with complex pharmacological effects including anti-cancer (Zhang L. X. et al., 2021). RSV shows potential benefits for COPD patients (Beijers et al., 2018). Studies have shown that RSV is associated with reducing inflammatory cell infiltration, improving the production of pro-inflammatory cytokines (TNF-α, IL-6, GM-CSF, IL-1β, and IL-8), and up-regulating antioxidant genes (SIRT1, PGC-1α, CAT, SOD1, and SOD2) in the treatment of COPD (Ma and Li, 2020; Wang et al., 2017). SIRT1 is a potential target of RSV in the treatment of COPD (Ma and Li, 2020). A mouse COPD model indicated that RSV maintains alveolar type-2 epithelial cell integrity by stimulating SIRT1 expression and promoting p53 destabilization (Navarro et al., 2017). RSV also restores steroid sensitivity of COPD lymphocytes and NKT-like cells and reduces systemic inflammatory responses by promoting SIRT1 expression (Hodge et al., 2020). RSV significantly increases PGC-1α and Nrf1 mRNA levels, mitochondrial mass, and coupled respiration through pharmacological activation of AMPK signaling to protect visual cortical neurons (Yu and Yang, 2010). RSV can upregulate antioxidant enzymes’ (SOD1, SOD2, and CAT) activities and restore reduced glutathione (GSH) biogenesis to protect astrocytes from oxidative damage (Bellaver et al., 2016).

AMPK/FOXO3 signaling is one of the vital pathways that regulate muscle atrophy; activated FOXO3 can induce the expression of atrophy-related genes, proteolysis, and muscle loss (Powers et al., 2012), and it is associated with intracellular ROS production (Akasaki et al., 2014) *via* NF-κB activation (Romanello et al., 2010). The ability of RSV to activate AMPK signaling to improve mitochondrial function and to increase mitochondrial biogenesis is dependent on SIRT1 (Price et al., 2012). These findings suggest that the development of new usages of RSV in COPD therapy might be *via* modulating mitochondrial function. However, the mechanisms of RSV anti-COPD effect *via* modulating mitochondrial activities need in-depth investigations.

### 3.4 Puerarin

Puerarin (PUE), an isoflavone derived from *Angelica* root, has various beneficial pharmacological functions (Jiang et al., 2022). The PI3K/Akt/mTOR pathway regulates autophagy, cell survival and differentiation, proliferation, and apoptosis (Hou et al., 2018), and it is involved in PM2.5-induced COPD (Mao et al., 2020). PUE can alleviate COPD by activating PI3K/Akt/mTOR signaling, inhibiting FUNDC1-mediated mitophagy and bronchial epithelial cell apoptosis (Liu J. et al., 2022).

PUE may serve as a novel and effective therapeutic agent for early-stage COPD. PUE can attenuate the acute smoking-induced infiltration of neutrophils and macrophages in mouse lung, leading to decreasing NF-κB signaling, and it also sensitively enhanced inflammatory mediator (TNF-α, COX-2, IL-6, MCP-1, and IL-8) expression; PUE also can suppress ROS production and upregulate NOX isoforms of small airway epithelia (Fang L. et al., 2022). It has been reported that PUE does not only increase mitochondrial antioxidant capacity, reduce overproduction of ROS, inhibit the expression of inflammatory factors and oxidative stress damage, and improve mitochondrial respiratory function and energy metabolism (Chang et al., 2021a) but also show mitochondrial protective properties in the treatment of nonalcoholic fatty liver disease (Wang S. et al., 2019), osteoarthritis (Wang et al., 2018), and diabetes (Chen et al., 2018).

These studies suggest that the mitochondrial pathway may be one of the mechanisms of using PUE in the treatment of COPD, but more research is needed.

### 3.5 Berberine

Berberine (BBR) is extracted from the bark and root of *Coptis chinensis* (Sun et al., 2022). A preliminary study indicates that BBR exhibits anti-inflammatory effects in treating chronic airway inflammatory diseases, including COPD (Tew et al., 2020). High-dose BBR attenuates CSE-induced airway inflammation by inactivating TGF-β1/Smads signaling (Wang W. et al., 2019).

In an *in vitro* study, BBR-loaded liquid crystal nanoparticles can reduce the expression of oxidative stress gene Nqo1 and inflammatory mediator TNF-α in bronchial epithelia and macrophages; it also inhibits p21 expression in bronchial epithelia (Paudel et al., 2022). BBR-loaded solid lipid nanoparticles (SLNs)-chitosan nanoparticles could dramatically ameliorate inflammation scores in lung tissues and reduce inflammatory cells (neutrophils and macrophages) and inflammatory cytokines (IL-1β, IL-6, IL-17, and TNF-α), thus improving the therapeutic anti-inflammatory impact of BBR against CS-induced airway inflammation in COPD rats (Sun et al., 2022).

BBR affects mitochondrial quality control and function by regulating the mitochondrial respiratory chain, oxidative stress, mitophagy, mitochondrial biogenesis and intracellular calcium concentration, and mitochondrial apoptosis (Fang X. et al., 2022). BBR can promote mitochondrial biogenesis (Yao et al., 2020) and restore autophagic flux (Hang et al., 2018) and mitochondrial energy homeostasis by regulating AMPK/PGC-1α signaling (Qin et al., 2020). BBR has also been shown to attenuate high-fat diet-induced muscle mitochondrial dysfunction by activating SIRT1/PGC-1α signaling, in which SIRT1 is involved in BBR-induced AMPK phosphorylation (Gomes et al., 2012). It is also reported that BBR protects the cardiac function of heart failure patients by upregulating PINK1/Parkin-mediated mitophagy (Abudureyimu et al., 2020) and attenuates myocardial ischemia/reperfusion impairment by regulating HIF-1α/BNIP3 signaling and enhancing mitophagy (Song et al., 2020). Furthermore, BBR inhibited NLRP3 inflammasome activation and ROS production by upregulating mitophagy, resulting in a reduction in lung inflammation in mice with influenza virus pneumonia (Liu H. et al., 2020). In addition, BBR can induce mitochondrial apoptosis, G0/G1 cell-cycle arrest, and inhibitory migration through modulating the PI3K/Akt and MAPK pathways of thyroid cancer cells (Li et al., 2017).

This suggests that BBR’s regulation of mitochondria may have an important place in new therapeutic strategies for COPD. Therefore, further studies are warranted to elucidate the detailed mechanism of the mitochondria-protective properties shown by BBR in the treatment of COPD.

### 3.6 Quercetin

Quercetin (QUE) is a plant flavanol (Zhu et al., 2018). It has been indicated that QUE protects COPD patients’ lymphocytes from 2-amino-3-methylimidazo [4, 5-f]quinoline (IQ)-induced DNA damage (Habas et al., 2018). QUE also reduces pulmonary inflammatory cell infiltration, oxidative stress, and lung function modification caused by CS exposure (da Silva Araújo et al., 2020), and it avoids emphysema changes (Araújo et al., 2022).

QUE has rhinovirus inhibitory properties *in vitro* and *in vivo*, including endocytosis, viral genome transcription, and viral protein synthesis (Ganesan et al., 2012). QUE effectively delayed lung disease progression in rhinovirus-infected COPD mice by reducing rhinovirus-induced pulmonary inflammatory cell accumulation, inhibiting mucus metaplasia and airway hyperresponsiveness (Farazuddin et al., 2018). QUE inhibits bronchial smooth muscle contraction induced by acetylcholine chloride and high K^+^ (Luo et al., 2018).

In addition, QUE modulates mitochondrial including mitochondrial quality control, mitochondria-mediated apoptosis pathways, MMP, oxidative respiratory chain and energy metabolism, and mitochondrial redox status etc. (de Oliveira et al., 2016). QUE has been shown to modulate mitochondrial properties in neuroprotective (Wang W. W. et al., 2021), cardioprotective (Chang et al., 2021b), enteroprotective (Vissenaekens et al., 2021), and hepatoprotective (Cai et al., 2021) effects. QUE reduces mtROS accumulation and subsequent NLRP3 assembly *via* enhancing mitophagy (Han et al., 2021). It is also known to inhibit NLRP3/caspase-1/GSDMD-N-mediated pyroptosis, maintain MMP, reduce mtDNA damage, and promote PGC-1α-mediated mitochondrial homeostasis, possibly through scavenging mtROS to protect liver cells from alcohol damage (Zhao et al., 2022). QUE was proven to upregulate SIRT1 expression, resulting in inhibiting NLRP3 activation and reducing neuroinflammation in aging mice (Li et al., 2021). It enhances the production of mitochondrial-related proteins (SIRT1, PGC-1α, and TFAM) expression (Ho et al., 2022) to inhibit apoptosis by modulating SIRT1/PGC-1α signaling (Tang et al., 2019); renal tubular epithelial cell senescence is associated with SIRT1/PINK1/Parkin activation (Liu T. et al., 2020). QUE can prevent sepsis-induced acute lung impairment and mitochondrial dysfunction by promoting the SIRT1/AMPK pathway (Sang et al., 2022). It can also restore amyloid-β-induced mitochondrial dysfunction by activating AMPK (Wang et al., 2014) and modulating the SIRT1/Nrf2/HO-1 pathway, leading to protecting the neurons in Alzheimer’s disease (Yu et al., 2020). It has been shown that QUE attenuates NF-κB signaling by inhibiting the HMGB1/TLR pathway (Li et al., 2016), and it inhibits NF-κB, TNF-α, IL-1β, IL-6, COX-2, and iNOS expression, reduces ROS levels, and increases SOD activity to attenuate Mn-induced oxidative stress and neuroinflammation (Bahar et al., 2017). Moreover, QUE was reported to downregulate mitochondrial apoptosis-related markers (BAX, Cyto c, cleaved caspase-3, and polymerase-1) and upregulate anti-apoptotic Bcl-2 expression, resulting in reduced Mn-induced apoptosis in SD rats (Bahar et al., 2017). However, on the other hand, QUE has also been reported to disrupt mitochondrial respiration between the ubiquinone pool and Cyto c that is associated with its cytotoxicity (Carrillo-Garmendia et al., 2022). It induces apoptosis and cell death of prostate cancer cells but not normal prostate epithelia *via* affecting mitochondrial integrity and interfering with ROS homeostasis (Ward et al., 2018).

However, few studies indicate the role of QUE-mediated mitochondrial function in the treatment of COPD. These results suggest that mitochondria may be the underlying mechanism of QUE in the treatment of COPD. However, further investigation needs to be carried out to test whether QUE has cytotoxic effects on normal cells.

### 3.7 Icariin

Icariin (ICA) is the main biologically active monomer of natural flavonoids extracted from *Epimedium brevicornum* Maxim (Li et al., 2015). It was found that ICA can reduce CSE-induced pro-inflammatory cytokine secretion and oxidative damage in bronchial epithelia, and it also has a positive effect on glucocorticoid resistance (Hu et al., 2020).

ICA attenuates LPS-induced acute lung inflammation *via* modulating the PI3K/Akt and NF-κB pathways (Xu et al., 2010). PI3K/Akt signaling plays an important role in the regulation of the Nrf2 pathway (Martin et al., 2004). ICA is considered a potential therapeutic agent of various diseases, such as cancers, neurodegenerative diseases, osteoporosis, and cardiovascular disease due to its ability to regulate the PI3K/Akt and Nrf2 signaling pathways (Verma et al., 2022). ICA promotes mitochondrial biogenesis in human nucleus pulposus cells by activating the PI3K/Akt and Nrf2 signaling pathways and inhibits hydrogen peroxide-induced mitochondria-mediated apoptosis (Hua et al., 2020). Icariside, a derivative of ICA, protects bone marrow mesenchymal cells from damage resulting from iron overload by regulating mitochondrial morphology and fission through the MAPK and PI3K/Akt/mTOR pathways (Yao et al., 2019).

Furthermore, ICA attenuates mitochondrial oxidative damage by enhancing SIRT1 activity and maintains mitochondrial homeostasis to protect cardiomyocytes from ischemia-reperfusion caused injury (Wu et al., 2018). ICA protects neurons from rotenone damage by upregulating SIRT3 and PGC-1α expressions (Zeng et al., 2019a). This protective effect may also be related to the restoration of autophagic flux by ICA *via* enhancing mitophagy-related proteins’ (LC3-II and Beclin-1) expression and inhibiting mTOR activation (Zeng et al., 2019b). ICA ameliorates ROS accumulation and mitochondrial dynamics disturbance of alcohol-impaired atrium by activating SIRT3/AMPK signaling (Yu et al., 2022). ICA can rescue high glucose-induced osteoblast differentiation *via* inhibiting mtROS and maintaining mitochondrial homeostasis (Liu J. et al., 2022). Additionally, ICA can enhance neuronal cell activity in a triple transgenic Alzheimer’s disease mouse model *via* maintaining mitochondrial key enzyme COX IV and promoting ATP generation (Chen et al., 2016). The ICA and β-azalone combination increases mitophagy-related proteins’ (Beclin-1, PINK1, and p-Parkin) expression to promote autophagosomes formation, leading to attenuating amyloid-β-induced nerve cell impairment (Wang N. et al., 2021).

These studies suggest that the mitochondrial pathway may be the mechanism for icariin to treat COPD and other mitochondria-associated diseases.

### 3.8 Paeoniflorin

Paeoniflorin (PF), a water-soluble monoterpene glucoside extracted from the root of *Paeonia suffruticosa*, has shown that the therapeutic effect of PF in a COPD rat model includes not only reducing airway inflammation and improving lung function but also improving oxidative stress condition by quenching ROS and upregulating antioxidant enzymes *via* Nrf2-dependent signaling (Lin et al., 2016).

PF can ameliorate ovalbumin-induced lung injury in a mouse model by restoring MMP, modulating mitochondrial function, and inhibiting pro-inflammatory cytokine release (Han et al., 2022). PF alleviates bortezomib-induced peripheral neuropathy (BiPN) by promoting Parkin-mediated mitophagy and decreasing IL-6 (Sun et al., 2022). In addition, PF can reduce the activities of caspase-9 and caspase-3, and it also inhibits the release of Cyto c in mitochondria, resulting in apoptosis inhibition (Liu Y. F. et al., 2022). PF can inhibit the expression of mitochondrial apoptotic proteins BAX, caspase-9, and caspase-3 and upregulate Bcl-2 by inhibiting JNK-related signaling pathways, resulting in apoptosis inhibition (Deng et al., 2022; Cong et al., 2019). PF can protect spiral ganglion neurons from cisplatin damage by reducing ROS and modulating the PINK1/BAD pathway (Yu et al., 2019). However, PF can promote apoptosis of synovial tissue by downregulating Bcl-2 and enhancing AMPK phosphorylation (Huang et al., 2021); it can promote mitochondrial biogenesis and improve TNFα-induced muscle atrophy *via* regulating TFAM, ERα, and Nrf1 expression (Park et al., 2022). It also improves mitochondrial dysfunction and oxidative stress induced by TNF-α by promoting the AMPK/SIRT1/PGC-1α pathway, (Li Q. et al., 2022), PF inhibits lipopolysaccharide-induced mitochondrial damage, and activation of the NLRP3 inflammasome of hepatocytes *via* the regulation of SIRT3/FOXO1a/SOD1 signaling (Li L. et al., 2022). Furthermore, it downregulates the ROS-NF-κB axis *via* suppressing NOX2/NOX4 and RAGE expression, which results in a decrease of the downstream HIF-1α/VEGF level, leading to protecting human umbilical vein endothelial cells from oxidative damage (Song et al., 2017) and inhibiting intracellular Ca^2+^ and calcium/calmodulin kinase II (CaMKII), which might show protective effects on mitochondria (Wang D. et al., 2013).

Even though few studies indicate that PF modulates mitochondrial function in COPD, the above pieces of evidence suggest that mitochondria may be the underlying molecular mechanism for PF in COPD treatment.

### 3.9 Paeonol

Paeonol (PAE) is one of the phenolic phytochemicals isolated from herbs such as *Dioscorea*, *Paeonia lactiflora*, and *P. suffruticosa*. PAE can reduce glutamate-induced apoptosis and neurotoxicity by suppressing Cyto c release and caspase-3 activation, as well as downregulating the ERK pathway (Wang et al., 2011). However, the mechanism of PAE in the treatment of COPD is yet to be understood. It has been shown that PAE inhibits hypoxia-mediated mitochondrial damage in pulmonary artery smooth muscle cells by decreasing ATP production, increasing ROS production, and enhancing mitochondrial morphological changes and polarization (Wang D. et al., 2019). PAE alleviates LPS-induced liver injury by improving mitochondrial function, maintaining MMP, reducing the expression of BAX and cleaved caspase-3, inhibiting superoxide production from mitochondria, and nuclear translocation of NF-κB (Xu et al., 2021).

PAE can reduce M1 macrophage polarization by inhibiting NLRP3 inflammasome, which results in decreasing inflammation levels in acute (Yuan et al., 2022). PAE can ameliorate streptozotocin-induced mtROS, TNF-α and IL-6, and MMP disturbance (Tayanloo-Beik et al., 2022). PAE derivatives have been shown to exert anti-inflammatory effects *via* suppressing TLR4/MyD88 signaling and inflammatory factor expression; they can also inhibit LPS-induced ROS production and restore the MMP of macrophages (Gong et al., 2022). In addition, PAE can upregulate PINK1/Parkin and BNIP3L/NIX autophagy signaling to protect retinal photoreceptor cells (Zhang D. et al., 2021). PAE can also promote apoptosis of hepatic stellate cells *via* inhibiting NF-κB signaling (Kong et al., 2013). PAE can induce apoptosis of cervical cancer cells that is associated with regulating the PI3K/Akt pathway to trigger mitochondrial apoptotic signaling (Du et al., 2022).

These studies suggest that PAE has potential mitochondrial protective activity, which supports the potential COPD therapy with PAE *via* modulating mitochondrial activities.

### 3.10 Hesperidin

Hesperidin (HES), a flavonoid glycoside present in citrus fruits, has been shown to possess antioxidant, anti-inflammatory, antiviral, anticancer, and neuroprotective properties (Garg et al., 2001; Xia et al., 2018). A recent study has indicated that HES ameliorates CSE-induced inflammation and oxidative stress by promoting SIRT1/PGC-1α/NF-κB signaling (Manevski et al., 2020). Derivatives of HES, such as hesperidin-3-O-methyl ether and hesperetin-5, 7, 3-O-trimethyl ether, inhibited airway hyperresponsiveness and inflammation in mouse models (Yang et al., 2012; Shih et al., 2020).

HES can improve mitochondrial malfunction during benzopyridine-induced lung carcinogenesis in a mouse model; for example, it upregulates antioxidant and TCA cycle enzymes, protects electron transport chains, and restores ATP levels (Kamaraj et al., 2011). The mechanism by which HES treats COPD is yet to be known.

HES alleviates 6-hydroxydopamine-induced degeneration of dopamine neurons by restoring mitochondrial respiratory chain complexes-I, -IV, and V as well as Na^+^-K^+^-ATPase activity and regulating caspase-3 and caspase-9 activities (Antunes et al., 2021). HES can protect against amyloid-β-induced neurotoxicity by suppressing VDAC1-mediated mitochondrial apoptosis signaling (Wang D. M. et al., 2013). HES reduces high glucose-induced apoptosis and oxidative damage of retinal pigment epithelia *via* scavenging ROS, decreasing Cyto c release, and inhibiting caspase-9/3 expression (Liu et al., 2018).

Furthermore, neohesperidin attenuated hepatic steatosis and insulin resistance that was associated with increasing PGC-1α-mediated mitochondrial biogenesis in a high-fat diet mouse model (Wang et al., 2020). HES can attenuate PM2.5-induced DNA damage, cell cycle arrest, and cellular senescence of human HaCaT keratinocytes via deactivating ROS/JNK signaling (Herath et al., 2022). HES can suppress high glucose-induced DNA damage and high mitochondrial calcium level to protect neuronal cells (Lim et al., 2022). HES also inhibits apoptosis and promotes cell viability by inactivation of ERK, JNK, and MAPK (Lim et al., 2022). Additionally, HES can reverse bupivacaine anesthesia-induced decreased MMP, mitochondrial apoptotic signaling, and HES can modulate the homeostasis between redox and inflammatory system (Wang T. et al., 2021). Furthermore, HES promotes DRP1-mediated mitophagy and improves impaired mitochondria in a functional dyspepsia rat model (Jia et al., 2022).

These studies suggest that the mitochondrial pathway might provide potential insights of HES for COPD therapy.

## 4 Conclusion

Although we have found some mechanisms and functions of the active monomers and combinations from herbal extractions that show mitochondrial protection ability in COPD therapy, the evidence from clinical studies that support the treatment of COPD with the natural compounds is still insufficient and remains inconclusive (according to ClinicalTrials.gov database, as accessed on 03/31/2024) (Table 1). Many natural compounds show effects on mitochondrial modification, but no COPD-related research has been performed that is worth considering in the future investigation. One such example is astragaloside IV (AS-IV), a small-molecule saponin extracted from *Astragalus membranaceus*, which improves mitochondrial activity in cortical neurons subjected to oxygen and glucose deprivation (OGD) by modulating PKA/CREB signaling, resulting in neuronal apoptosis inhibition by regulating mtROS and ATP production (Xue et al., 2019; Ryu et al., 2005). This might have potential effects on regulating COPD mitochondrial homeostasis.

**TABLE 1:** Selected natural extract compounds from bench to clinical applications.

No.	Compound	Structure	Source	Disease	Clinical trials
1	Curcumin (CUR)	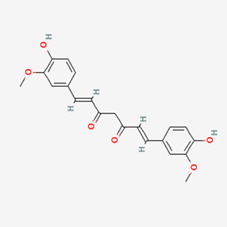	Turmeric (*Curcuma longa*)	COPD (Zhang et al., 2016) Cancer Inflammation	NCT03769766 NCT03980509 NCT01859858 NCT02598726 NCT05975866 NCT01514266
2	Ginsenosides (GS)	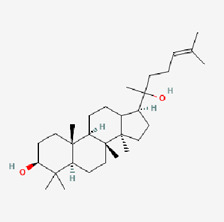	*Panax ginseng*	COPD (Chen et al., 2008; Lin et al., 2022) Metabolic syndrome (Zhou et al., 2019b)	NCT02034136
3	Resveratrol (RSV)	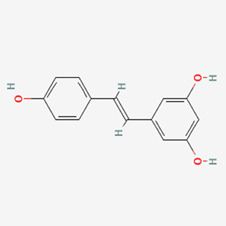	Fruits Vegetables	Type-2 diabetes Cardiovascular disease COPD (Beijers et al., 2018) Inflammation	NCT06131918 NCT03819517 NCT02245932 NCT06020313 NCT01564381 NCT02244879 NCT02433925 NCT01492114
4	Puerarin (PUE)	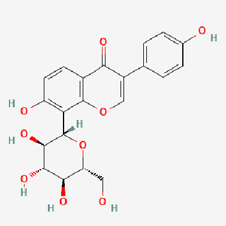	*Angelica* (root)	COPD (Liu et al., 2022b) Heart health Metabolism syndrome (Chen et al., 2018) Rheumatoid arthritis (Wang et al., 2018) Nonalcoholic fatty liver disease (Wang et al., 2019a)	NCT03676296 NCT02219191 NCT02254655
5	Berberine (BBR)	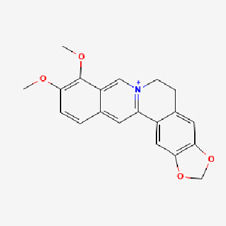	*Coptis chinensis* (bark and root)	COPD (Sun et al., 2022) Diabetes Cardiovascular disease (Song et al., 2020) Inflammation/COVID-19 (Liu et al., 2020b) Cancer (Li et al., 2017)	NCT02808351 NCT04698330 NCT05105321 NCT04479202 NCT04434365
6	Quercetin (QUE)	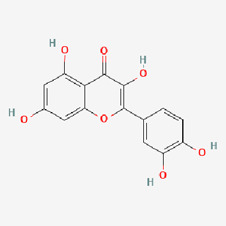	Fruits, vegetables, and many others	COPD (Habas et al., 2018) Alzheimer’s disease (Yu et al., 2020) Inflammation (Bahar et al., 2017) Cancer (Ward et al., 2018)	NCT06003270 NCT03989271 NCT04063124 NCT05371340 NCT00003365 NCT01912820
7	Icariin (ICA)	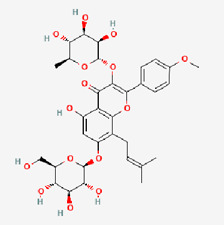	*Epimedium brevicornum* Maxim	Inflammation (Xu et al., 2010) Respiratory system (Hu et al., 2020) Neuro disease (Chen et al., 2016) Osteoporosis (Verma et al., 2022) Cardiovascular disease (Verma et al., 2022) Cancer (Verma et al., 2022) Mental health	NCT02112123 NCT01979133
8	Paeoniflorin (PF)	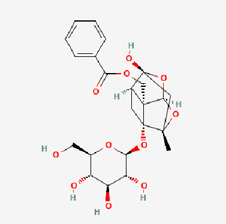	*Paeonia suffruticosa* (root)	COPD (Lin et al., 2016) Inflammation (Han et al., 2022) (Li et al., 2022b) Auto-immune hepatitis	NCT02878863 (withdrawn)
9	Paeonol (PAE)	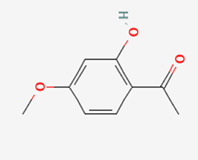	*Dioscorea* *Paeonia lactiflora* *Paeonia suffruticosa*	Inflammation (Yuan et al., 2022) Cancer (Du et al., 2022) Fibrosis (Kong et al., 2020) Knee osteoarthritis	EU clinical trial: 2020–000249–14
10	Hesperidin (HES)	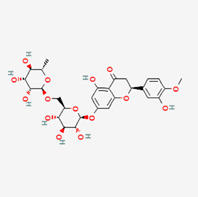	Citrus fruits	Neuro disease (Garg et al., 2001; Xia et al., 2018) Respiratory system (Manevski et al., 2020) Bone health Metabolic syndrome (Wang et al., 2020) Inflammation (Wang et al., 2021e) COVID-19 symptoms	NCT00330096 NCT03734835 NCT03734874 NCT04715932

Note: 1: The structure of compounds was obtained from the NIH website (https://pubchem.ncbi.nlm.nih.gov). 2: For more detailed information about the selected clinical trials, please visit the websites (ClinicalTrials.gov and https://pubchem.ncbi.nlm.nih.gov).

Impaired mitochondria have different appearances and effects on different types of cells, stages of growth, and diseases. All these different conditions should be considered to maximize the efficiency of administration. Additionally, the effects of natural compounds on various mitochondrial targets should be well investigated and considered. Based on the concerns mentioned above, a comprehensive and systematic investigation strategy on herb extractions should be carried out to promote their use for mitochondrial related other diseases.
